# Intercropping Lettuce with Alfalfa Under Variable Nitrate Supply: Effects on Growth Performance and Nutrient Dynamics in a Vertical Hydroponic System

**DOI:** 10.3390/plants14132060

**Published:** 2025-07-05

**Authors:** Luis D-Andrade, Nivia Escalante-Garcia, Ernesto Olvera-Gonzalez, Francesco Orsini, Giuseppina Pennisi, Felix Vega de Luna, Hector Silos-Espino, Cinthia Najera

**Affiliations:** 1 Laboratorio de Iluminación Artificial, Tecnológico Nacional de México/IT de Pabellón de Arteaga, Carretera a la Estación de Rincón Km. 1, Aguascalientes 20670, Mexico; luis.da@pabellon.tecnm.mx; 2 Department of Agricultural and Food Sciences, University of Bologna Alma Mater Studiorum, Viale Giuseppe Fanin 44, 40127 Bologna, Italy; f.orsini@unibo.it (F.O.); giuseppina.pennisi@unibo.it (G.P.); 3 UMR7141, Institut de Biologie Physico-Chimique, 75005 Paris, France; 4 Laboratorio de Bioprocesos, Tecnológico Nacional de México, Instituto Tecnológico del Llano, Km. 18, Carr. Ags.-S.L.P., El Llano, Aguascalientes 20330, Mexico; hector.se@llano.tecnm.mx; 5 Department of Agronomy, University of Almería, 04120 Almería, Spain; cnajera4@ual.es

**Keywords:** intercropping, nitrate, hydroponics, lettuce, alfalfa, nutrient uptake, root zone, pH, electrical conductivity (EC), vertical farming

## Abstract

Vertical farming systems offer an efficient solution for sustainable food production in urban areas. However, managing nitrate (NO_3_^−^) levels remains a significant challenge for improving crop yield, quality, and safety. This study evaluated the effects of nitrate availability on growth performance, nutrient uptake, and water use efficiency in a vertical hydroponic system that intercropped lettuce (*Lactuca sativa*) with alfalfa (*Medicago sativa*). The experiment was conducted in a controlled vertical hydroponic system using Nutrient Film Technique (NFT) channels, with nitrogen levels set at 0, 33, 66, 100, and 133% of the standard concentration. The results indicated that the intercropping treatment with 66% nitrate (IC-N_66%_) improved water use efficiency by 38% and slightly increased leaf area compared to the other intercropping treatments. However, the control group, which consisted of a monoculture with full nitrate supply, achieved the highest overall biomass. Ion concentrations, including nitrate, calcium, magnesium, and micronutrients, were moderately affected by the intercropping strategy and nitrate levels. These findings suggest that moderate nitrate input, combined with nitrogen-fixing legumes, can enhance resource efficiency in hydroponic systems without significantly compromising yield. These findings offer a promising framework for incorporating legumes into hydroponic systems, minimizing the need for synthetic inputs while maintaining yield. These results support the use of agroecological intensification strategies in highly efficient soilless systems.

## 1. Introduction

Vertical farms (VFs) represent an innovative solution that transforms traditional food production by efficiently adapting to densely populated urban environments [1]. These agricultural systems utilize multi-layered structures in controlled environments and incorporate advanced technologies such as hydroponics, LED lighting, and automated environmental control systems. This integration allows for optimized space usage, increased crop yields, and enhanced resource efficiency, enabling the continuous production of high-value, perishable crops like leafy vegetables and herbs, especially in urban settings [2]. However, the success of vertical farming depends not only on its engineering design but also on its ability to improve key physiological processes in plants under controlled conditions. Factors such as nutrient management—particularly nitrogen dynamics—light quality and intensity, temperature, and water availability can be carefully regulated in these systems [3]. This opens up new opportunities to enhance photosynthetic efficiency, reduce abiotic stress, and optimize nutrient uptake in species such as lettuce. Nitrogen is an essential element for the biosynthesis of amino acids, nucleic acids, and chlorophyll, making it a primary limiting factor in plant growth. However, inadequate nitrogen supply practices—particularly excessive or poorly timed application of nitrate (NO_3_^−^)—can induce physiological alterations in crops grown under both conventional and advanced hydroponic systems [4,5,6]. In vertical farms, nitrogen availability and light intensity influence photosynthesis and nitrate accumulation. For example, lettuce grown aeroponically under low light accumulated up to 75% more nitrates due to limited reduction into proteins [7]. Similar patterns were seen in *Brassicaceae* microgreens under suboptimal LED light conditions [8].

In NFT (Nutrient Film Technique) systems, the continuous flow of nutrient solutions means that nitrate (NO_3_^−^) has an impact on ion uptake, root growth, and nutrient distribution within the plant. When managed correctly, nitrate enhances the absorption of essential elements such as potassium, calcium, and magnesium. However, if nitrate levels are excessively high or unbalanced, it can lead to nutrient competition or decreased absorption efficiency. Therefore, precise control of nitrate levels is crucial for optimal plant health [9].

Previous research has demonstrated that altering nitrate (NO_3_^−^) concentrations in nutrient solutions can significantly impact biomass production, photosynthetic efficiency, nitrate reductase activity, and the uptake of essential ions such as Ca^2+^, K^+^, and Mg^2+^. These factors are all crucial for the organoleptic and nutritional quality of leafy vegetables like lettuce [10,11,12,13]. However, most studies have focused on cultivation systems other than NFT, typically examining static or batch hydroponic conditions. This indicates a need to verify whether similar physiological responses occur in continuous flow. In modified NFT setups, it was found that suspending nitrogen fertilization 2 to 4 days before harvest could reduce foliar nitrate accumulation by 29 to 58% without affecting yield [14]. Additionally, dynamic NO_3_^−^ dosing strategies implemented in aeroponic systems have achieved reductions of up to 92% in leaf nitrates based on varying light conditions [7].

A complementary agroecological strategy to improve nitrogen dynamics includes intercropping legumes such as *Medicago sativa* (alfalfa). These legumes form symbiotic relationships with nitrogen-fixing bacteria such as *Rhizobium* spp. and plant growth-promoting bacteria (PGPB). These interactions enhance biological nitrogen fixation (BNF), improve nutrient uptake, and decrease the reliance on synthetic nitrogen fertilizers. This approach has proven particularly effective under controlled conditions, where rhizospheric enhancement can optimize lettuce performance in hydroponic systems [15,16,17,18,19,20]. According to Guerchi [21], intercropping alfalfa with *Hordeum marinum* under saline conditions (0 and 150 mM NaCl) resulted in superior biomass accumulation and enhanced nutrient uptake specifically in *Hordeum marinum*. This improvement was accompanied by a reduction in soil pH and electrical conductivity, suggesting that alfalfa contributed to rhizosphere stabilization and ionic balance through biological interactions. In soilless systems, NFT allows for precise dosing of nitrates and effective control of variables such as pH and electrical conductivity (EC) [22,23].

These benefits position NFT as a crucial technology for short-cycle crops with high nutrient demands, such as lettuce. In greenhouse conditions, the intercropping of lettuce with alfalfa has been studied for its potential to enhance biomass accumulation, improve nutrient use efficiency, and increase postharvest quality [24]. A randomized field experiment conducted under controlled conditions demonstrated that incorporating alfalfa into the soil, along with solid nitrogen fertilization at rates of 200–300 kg per hectare, significantly improved yield [24]. This approach also resulted in a reduction in nitrate (NO_3_^−^) content in the lettuce sap and optimized the uptake of potassium and calcium in lettuce.

Recent studies indicate that the concurrent cultivation of alfalfa and lettuce under conditions of heavy metal stress, specifically lead (Pb) and cadmium (Cd), significantly enhances the antioxidant response in lettuce [25]. Furthermore, findings demonstrate that alfalfa modifies the rhizosphere microenvironment of lettuce within hydroponic greenhouse systems [26]. Soilless cultivation of lettuce presents certain phytosanitary challenges. Pathogens such as *Pythium* spp., *Fusarium* spp., *Erwinia*, and *Pseudomonas* thrive in controlled environments. One study reported an increased risk of human pathogens like *Salmonella* being absorbed into hydroponic lettuce if factors such as pH, aeration, and water quality are not properly managed [5]. To reduce this risk, incorporating legumes such as alfalfa is suggested, as they promote microbial diversity and enhance the rhizosphere environment shared with neighboring crops like lettuce.

Vertical farms (VFs) exemplify the highest level of technological integration in agriculture. By combining LED lighting, automated CO_2_ control, and precise nutrient management, these farms enable unparalleled accuracy in studying interspecific interactions. Singh et al. [27] reported that supplementing CO_2_ in NFT vertical farms increased both the yield and the mineral content of lettuce. This finding confirms that such an environment serves as an ideal model for evaluating complex interactions, like the alfalfa–lettuce intercropping, under controlled conditions. Investigations have documented the effects of nitrogen on foliar morphophysiological parameters. Adequate nitrogen availability has been shown to increase specific leaf area (SLA) and enhance photosynthetic efficiency [28,29].

Advancements in nutrient management for hydroponic systems have been significant; however, the integration of legumes into vertical hydroponic systems remains largely unexplored. This study aimed to evaluate the effects of nitrate availability in a lettuce–alfalfa intercropping system on biomass production, nutrient uptake, ion distribution, and root-zone physicochemical responses within a closed-loop Nutrient Film Technique (NFT) system. The findings are anticipated to enhance nitrate management, contribute to strategies for reducing fertilizer use, and improve resource efficiency in sustainable vertical farming.

## 2. Results

### 2.1. Nutritive Solution Dynamics

#### 2.1.1. Water Consumption

**Figure 1:**
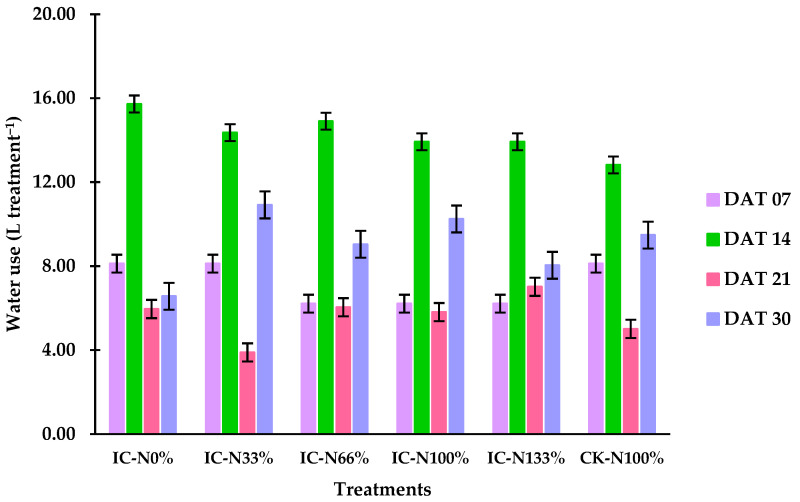
Water consumption by treatment across four sampling dates (7, 14, 21, and 30 DAT). Bars represent mean values ± standard deviation (*n* = 3).

Figure 1 illustrates the water consumption patterns across treatments during the four-week cultivation period, measured in days after transplanting (DAT). Treatments IC-N_0%_ and IC-N_33%_ exhibited slightly higher water retention, averaging 41.3 L. In contrast, IC-N_66%_, IC-N_100%_, and IC-N_133%_ showed slightly higher water retention, averaging 41.3 L. Among all treatments, IC-N_0%_ exhibited the highest overall consumption, reducing its water volume to 23.7 L, while IC-N_100%_ and IC-N_133%_ retained the most water, both ending at 27.4 L. By the end of the experiment, water levels in IC-N_0%_ and IC-N_33%_ were the lowest, with 11.1 L and 10.2 L, respectively, compared to CK-N_100%_, which remained at 12.1 L. Notably, IC-N_33%_ experienced the sharpest decline in water availability, while IC-N_133%_ retained the highest final volume, indicating a potential dose-dependent effect related to treatment application.

#### 2.1.2. pH Dynamics

The pH dynamics observed across the treatments, as detailed in Table 1, exhibited fluctuations over the four-week period. Adjustments were made to maintain optimal pH ranges of 5.5 to 6.5 for both low- and medium-salinity hydroponic treatments. The CK-N_100%_ group consistently acidified the nutrient solution by the second, third, and fourth weeks. Conversely, significant increases in pH were noted in the treatments IC-N_0%_ (6.87), IC-N_33%_ (7.36), IC-N_66%_ (6.92), and IC-N_133%_ (7.15). The IC-N_100%_ treatment experienced a smaller pH increase, reaching 6.25. The alfalfa treatments typically stabilized between 6.0 and 6.5, except for IC-N_33%_, which remained slightly elevated at 6.46. Notably, the IC-N_100%_ treatment required minimal adjustments.

#### 2.1.3. Electrical Conductivity (EC)

**Figure 2:**
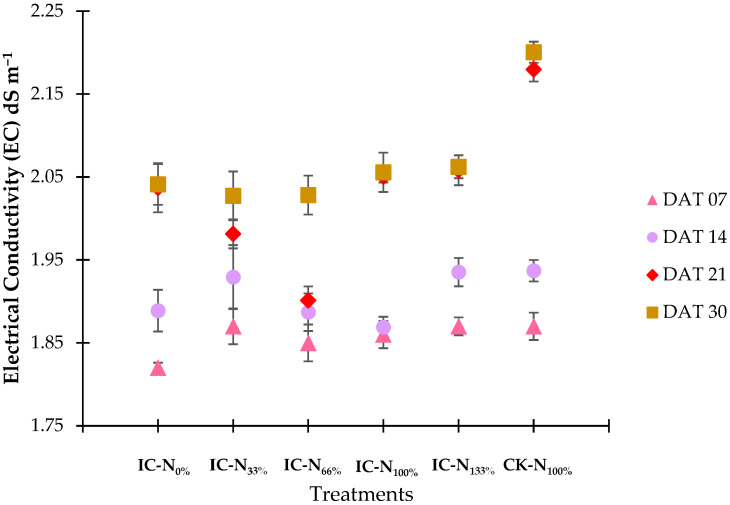
Nutrient solution EC by treatment across DAT-based timepoints.

Figure 2 illustrates that the electrical conductivity (EC) values consistently increased across all treatments, reflecting the accumulation of ions as water was consumed. By the fourth week of the experiment, the control group recorded the highest EC at 2.20 dS m^−1^. In contrast, treatments IC-N_0%_ and IC-N_33%_ displayed lower final EC values of 2.04 dS m^−1^, which corresponded with their higher levels of water consumption (as noted in Section 2.1.1). Treatments IC-N_100%_ and IC-N_133%_ achieved an EC of 2.06 dS m^−1^, indicating effective nutrient uptake. Despite these rising trends, the EC levels remained within the expected range throughout the experiment, negating the need for adjustments and ensuring stable nutrient availability.

### 2.2. Plant Growth and Biomass Accumulation

Table 2 displays the data on the accumulation of fresh and dry biomass across different treatments. An increase in both fresh and dry mass was observed with higher levels of nitrogen fertilization. The total fresh weight of the plants significantly increased, rising from 37.21 g in treatment IC-N_0%_ to 136.75 g in treatment IC-N_133%_, while the control group recorded the highest weight at 147.83 g. Notably, the leaf fresh weight in treatments with elevated nitrogen levels reached 92.9% in IC-N_133%_ compared to the control, whereas IC-N_0%_ had the lowest fresh weight percentage, at 77.9%, but a relatively higher dry weight percentage of 22.1%. This pattern suggests that increased nitrogen availability enhances water accumulation in plant tissues. Furthermore, the mass lost during drying followed a similar trend, being significantly lower in IC-N_0%_, at 12.84 g, and highest in the control group, at 104.71 g, which further indicates improved tissue hydration due to nitrogen fertilization. Although IC-N_66%_ performed better than other intercropping variants, CK-N_100%_ still exhibited the highest total fresh and dry biomass, confirming the advantages of full nitrate supply in monoculture.

**Figure 3:**
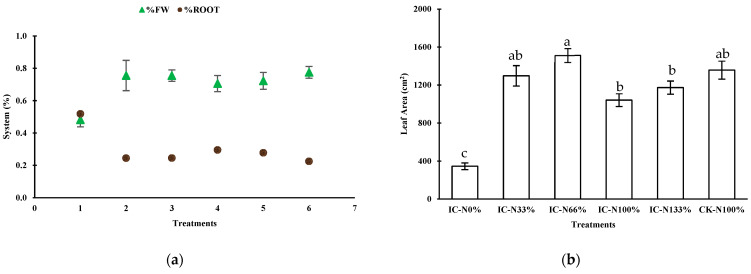
Effects of nitrate availability on biomass allocation and leaf development in lettuce. (**a**) Proportion of fresh weight (%FW) versus root weight (%Root) across treatments. (**b**) Leaf area variation per treatment at 30 days after transplanting. Different letters in b indicate significant differences (Tukey, *p* ≤ 0.05).

Figure 3 illustrates the changes in root mass and the proportion of root biomass across different treatments. The root mass consistently increased (3a), starting at 19.27 g in treatment IC-N_0%_ and rising to 37.97 g in IC-N_133%_. Meanwhile, CK-N_100%_ recorded a root mass of 33.23 g. This trend suggests that a higher availability of nitrate (NO_3_^−^) enhances the plant’s ability to absorb water and nutrients. However, the percentage of root biomass (% root) relative to the total plant mass decreased as nitrate levels increased. It fell from 52% in IC-N_0%_ to 28% in IC-N_133%_, reaching a low of 22% in CK-N_100%_. This decline indicates that under conditions of low nitrogen availability, plants allocate a greater proportion of their biomass to root development to maximize nutrient acquisition. In contrast, higher nitrate levels encourage a shift in biomass allocation toward the shoots, which increases the percentage of aboveground fresh weight (%FW).

In the treatment IC-N_0%_, plants had the lowest number of leaves, averaging 16.50. In contrast, the treatment IC-N_66%_ produced the highest count, with an average of 30.33 leaves. The control treatment CK-N100% closely followed, averaging 28.67 leaves, while IC-N_33%_ had an average of 29.00 leaves. The total leaf area increased with the application of nitrate. IC-N_0%_ had a leaf area of 344.90 cm^2^, while the nitrate treatments ranged from 1041.95 cm^2^ in IC-N_100%_ to 1512.62 cm^2^ in IC-N_66%_. The control group achieved a leaf area of 1357.75 cm^2^. Leaves from IC-N_0%_ exhibited a lower specific leaf area (SLA) of 70.49 cm^2^/g dry weight (DW), indicating that these leaves were denser and thicker, likely due to nitrate deficiency. In contrast, the nitrogen-fertilized treatments showed SLA values ranging from 138.17 to 202.2 cm^2^/g DW, suggesting that these leaves were thinner and broader, optimizing light capture under increased nitrogen availability. Water use efficiency (WUE) was lower in IC-N_0%_, measured at 1.572 g dry weight (DW) per liter of water (H_2_O), compared to the fertilized treatments. The CK-N_100%_ treatment achieved the highest efficiency, at 3.264 g DW per liter of H_2_O, followed by IC-N_66%_, with 2.811 g DW per liter of H_2_O.

### 2.3. Accumulation of Mineral Elements in Plant Tissue

All macronutrient and micronutrient concentrations presented in this section are expressed in milligrams per gram of dry weight (mg g^−1^ DW). Table 3 summarizes the macronutrient content across the different treatments. A significant difference was observed in the IC-N_0%_ treatment, where the levels of calcium (Ca), potassium (K), phosphorus (P), magnesium (Mg), sulfur (S), and nitrogen (N) were notably lower per unit of dry biomass compared to the other treatments. In contrast, the remaining treatments showed macronutrient concentrations that were comparable to those of the control group. Nitrogen (N) content displayed clear statistical differences, with the lowest value recorded in the IC-N_0%_ treatment (12.8c). Fertilized treatments had significantly higher nitrogen levels, peaking in the IC-N_100%_ treatment (43.8a). The IC-N_133%_ (41.3ab) and CK-N_100%_ (40.4ab) treatments did not significantly differ from each other, suggesting that nitrogen uptake may plateau beyond a certain availability threshold. Phosphorus (P) content also varied significantly across treatments. The IC-N_0%_ treatment had the lowest value (3.8d), while the highest concentrations were found in the IC-N_133%_ (11.3a) and CK-N_100%_ (11.2a) treatments. Intermediate values were recorded in the IC-N_33%_ (10.1b) and IC-N_66%_ (8.6c) treatments, indicating that phosphorus uptake improves with increased nitrogen availability, up to a saturation point.

Potassium (K) showed a significant response to fertilization, increasing from 37.3 in the IC-N_0%_ treatment to a maximum of 85.2 in the IC-N_33%_ treatment. However, higher treatments (IC-N_66%_, IC-N_100%_, and IC-N_133%_) exhibited lower potassium levels, measuring 77.2, 79.0, and 82.6, respectively. The CK-N_100%_ treatment also recorded a similar value, of 77.6. These results indicate that potassium uptake is more efficient at moderate levels of nitrogen fertilization, as no significant increases in potassium were observed at higher dosages. Calcium (Ca) recorded its lowest value in IC-N_0%_, with a concentration of 6.3, and reached its peak in IC-N_33%_, with 13.2. The subsequent treatments showed similar calcium values, ranging from 11.1 to 11.8, with no significant differences among them. This suggests that calcium uptake stabilizes after reaching a certain level of available nitrogen.

Magnesium (Mg) showed a similar trend in its values, with the lowest recorded at IC-N_0%_ (2c) and the highest values observed in IC-N_33%_ and IC-N_100%_, measuring 3.8a and 3.7a, respectively. The values in the other treatments were intermediate, with measurements of 3.3b in IC-N_66%_ and 3.1b in CK-N_100%_. This pattern suggests that nitrogen availability enhances magnesium uptake to some extent. Regarding sulfur (S), the lowest value was also noted in IC-N_0%_ (1.5d). This value increased with fertilization, peaking at IC-N_100%_ (3a). Although the values in IC-N_133%_ and in the control treatment were slightly lower (2.9a and 2.8ab, respectively), there was no significant difference compared to IC-N_100%_. This indicates that sulfur responds positively to nitrogen up to a certain level.

Micronutrient levels varied significantly across different nitrogen fertilization treatments. Iron (Fe) concentrations were lowest in the IC-N_0%_ treatment, measuring 19.5d, but increased with higher nitrogen fertilization, reaching the highest level of 48.3a in the control group. The IC-N_33%_, IC-N_100%_, and IC-N_133%_ treatments displayed intermediate iron values of 39.1b, 39.2b, and 42b, respectively. There were no significant differences among these three treatments. This suggests that while nitrogen availability enhances iron uptake, other factors may limit its absorption beyond a certain threshold.

Zinc (Zn) concentrations were also lowest in the IC-N_0%_ treatment, at 65.3c, reaching a peak of 262a in the IC-N_100%_ treatment. The zinc levels for IC-N_33%_ and IC-N_100%_ were similar, recorded at 241ab and 244ab, respectively. However, IC-N_133%_ and the control group exhibited lower levels, at 223b and 234b. This indicates that zinc uptake is higher at intermediate fertilization levels but tends to decrease at elevated levels. Manganese (Mn) had its lowest concentration in IC-N_0%_ (239d) and peaked in IC-N_66%_ (477a). The IC-N_100%_ treatment displayed an intermediate value of 419b, while treatments IC-N_33%_, IC-N_133%_, and CK-N_100%_ fell within a similar range of 373c to 374c. This suggests that nitrogen availability enhances manganese uptake up to the IC-N_66%_ level, but beyond that, the uptake begins to decline.

Copper (Cu) exhibited a pattern similar to that of other nutrients, with its lowest value recorded in IC-N_0%_ (3.16d) and peaking in CK-N_100%_ (12.9a). The treatments IC-N_100%_ and IC-N_133%_ showed intermediate values of 11.5b and 11.2b, respectively, while IC-N_33%_ and IC-N_66%_ had notably lower values, at 9.97c and 9.63c. These results suggest that copper uptake improves with fertilization; however, the control group displayed even higher uptake, likely due to other environmental factors. Boron (B) reached its highest level in IC-N_33%_ (42.5a), with similarly high values observed in IC-N_100%_ (42.3a). In contrast, IC-N_133%_ showed a decrease (34.8bc), and IC-N_0%_ recorded the lowest value (30.6d). This indicates that boron uptake is most effective at moderate levels of nitrogen fertilization but tends to decline when nitrogen is in excess.

Nickel (Ni) showed a pattern that differs from most other nutrients. It reached its highest value in IC-N_0%_ (5.52a) but experienced a decline in the fertilization treatments, dropping to its lowest level in IC-N_66%_ (0.16c). Although CK-N_100%_ recorded a higher value (0.64b), it remained lower than the value in IC-N_0%_. This suggests that the availability of nitrogen decreases nickel uptake, likely because the presence of sufficient available nitrogen reduces the demand for this element. Molybdenum (Mo) uptake varied with different nitrogen fertilization levels. The highest uptake was observed in IC-N_33%_, with a value of 0.12a. In contrast, the lowest uptake occurred in IC-N_100%_, which recorded a value of 0.05e. The other treatments had intermediate values: CK-N_100%_ was at 0.1b, and IC-N_133%_ was at 0.09c. This suggests that high levels of nitrogen fertilization adversely affect molybdenum uptake, likely due to competition with other anions present in the nutrient solution. This pattern supports the hypothesis that nitrogen levels modulate ionic uptake both quantitatively and selectively, as demonstrated by CK-N_100%_, which showed maximal nitrogen and phosphorus accumulation.

### 2.4. Energy Consumption

Table 4 displays the monthly energy consumption of the main subsystems in the vertical farming setup. Among all the components, the lighting system was the most energy-intensive, consuming 293.8 kWh per month, which accounted for 47.88% of the total energy demand, even with the use of high-efficiency LED fixtures. Following this, the temperature control subsystem consumed 206.1 kWh per month, representing 33.58%, highlighting its critical role in maintaining optimal growing conditions. The irrigation system, which includes pumps and nutrient dosing mechanisms, accounted for 99.7 kWh per month (16.25%). In contrast, CO_2_ injection had a relatively minor consumption of 2.7 kWh per month (0.45%). Lastly, the control and monitoring subsystem required 11.3 kWh per month, making up 1.84% of the total energy consumption, which reflects the essential energy needed for automation and data logging within the production environment.

While energy use was not directly manipulated, these data emphasize the excessive burden of artificial lighting in vertical farms, a crucial factor when assessing sustainability.

## 3. Discussion

### 3.1. Nitrate Availability and Lettuce Growth Performance

The findings show that the availability of nitrate (NO_3_^−^) in a vertical hydroponic Nutrient Film Technique (NFT) system has a significant impact on lettuce growth, particularly when intercropped with alfalfa. Amidst the different intercropping treatments, the IC-N66% treatment produced the highest biomass, averaging 75.3 g per plant. This treatment outperformed both the nitrate-deficient treatment (IC-N_0%_) and the fully fertilized intercropped treatment (IC-N_100%_). However, the control variant (CK-N_100%_), which was a fully fertilized monoculture, achieved the highest overall fresh and dry biomass. These findings indicate that symbiotic intercropping can maintain productive growth with reduced nitrogen inputs while limiting nitrate accumulation in edible plant parts. This is consistent with previous research showing that high levels of nitrate can decrease nitrate reductase (NR) activity and protein synthesis, leading to increased nitrate accumulation in leafy vegetables [7,13,49]. As noted by Martínez-Moreno et al. [12], the efficiency of nitrate use depends not only on the levels of supply but also on environmental factors such as crop associations, light quality, and the developmental stage of the plants.

### 3.2. Nitrate Accumulation and Food Safety

Leaf nitrate concentrations were significantly lower under the IC-N_66%_ treatment (1120 mg kg^−1^) compared to the IC-N_100%_ treatment (1580 mg kg^−1^). Integrating biological nitrogen fixation with a reduced supply of nitrates can effectively moderate nitrate uptake and assimilation in lettuce. While the reductions observed were modest, they still comply with the nutritional safety standards established by the European Union. Additionally, these findings support previous studies indicating that excessive availability of nitrates may inhibit the activity of nitrate reductase (NR) and result in increased nitrate accumulation in leaf tissues [7,13,49].

### 3.3. Water Use Efficiency and Leaf Area

Water use efficiency (WUE) peaked in IC-N_66%_, reaching 2.811 g DW L^−1^, which represents a 38% increase compared to the monoculture control. This improvement highlights the synergistic effects of moderate nitrate input and biological nitrogen fixation in enhancing water conservation and metabolic performance. Similar gains in WUE (30–40%) have been reported in legume-based hydroponic systems [50], where moderate nitrate availability is known to boost photosynthetic activity. In this study, the specific leaf area (SLA) also significantly increased in IC-N_66%_, rising by 43% compared to IC-N_0%_. This indicates improved light capture and photosynthetic efficiency, consistent with nitrate-responsive morphological adjustments influenced by photoreceptor signaling [51]. These outcomes highlight the agronomic benefits of fine-tuning nitrogen supply to enhance both growth and water management in high-yield leafy vegetables under indoor systems [52,53].

### 3.4. Root-Zone Physicochemical Conditions (pH and EC)

Intercropping with alfalfa resulted in improved stability in the conditions of the rhizosphere. In the IC-N_66%_ treatment, the pH remained within the optimal range of 6.1 to 6.7, while the electrical conductivity (EC) decreased from 2.1 to 1.5 mS cm^−1^. This decrease indicates enhanced nutrient uptake and reduced salt accumulation. The favorable modulation of the root-zone environment is likely driven by legume root exudates, which enhance ionic balance and nutrient assimilation efficiency [54,55]. In contrast, the monoculture control (CK-N_100%_) exhibited suboptimal conditions, with a pH drop to 5.3 and the EC rising to 2.2 mS cm^−1^, suggesting accumulation of unassimilated ions—possibly potassium or sulfate—in the absence of alkalinizing exudates from alfalfa roots [56]. Although the pH was partially buffered, EC levels were not corrected during the cycle, emphasizing the need for continuous nutrient monitoring in closed-loop hydroponic systems [57].

### 3.5. Ion Uptake and Nutrient Balance

Moderate nitrate availability (IC-N_66%_) improved the uptake of essential micronutrients, particularly zinc and manganese. Conversely, in conditions with high nitrate levels, the concentrations of these micronutrients decreased by 33% and 36%, respectively. This reduction is likely due to ionic antagonism and dilution effects, as previously noted [10,50]. A similar pattern was observed for nickel, which showed decreased uptake in nitrate-rich treatments. This aligns with the findings of Jokinen et al. [49], who reported that sufficient levels of macronutrients can reduce the demand for specific micronutrients. These observations emphasize the significance of maintaining ionic balance in hydroponic systems. The interactions between nutrients not only affect plant growth but also have a direct impact on the nutritional quality of the edible tissues.

### 3.6. Biological Interaction and Rhizospheric Resilience

The symbiotic relationship with alfalfa provided clear physiological benefits. Under stress conditions, antioxidant enzymes such as catalase (CAT) and superoxide dismutase (SOD) increased by 25–40% in intercropped treatments, indicating improved tolerance to oxidative stress. These results align with previous research showing that plant growth-promoting rhizo-bacteria (PGPR) enhance nitrogen uptake and stress resilience in companion crops [58,59]. This highlights the ecological importance of functional intercropping in hydroponic agriculture [60].

### 3.7. Implications for Sustainable Vertical Farming

The IC-N_66%_ treatment showed the best overall performance by combining high productivity with improved efficiency in water and energy use, reduced nitrate accumulation, and enhanced nutrient uptake—all achieved without a heavy reliance on synthetic inputs. These findings align with the research of Miserocchi and Franco [61], who reported that optimized nutrient and lighting regimes can decrease energy consumption from 10–18 kWh per kilogram to just 3–7 kWh per kilogram. This highlights the importance of integrated strategies for improving energy efficiency in vertical farming systems. Additionally, incorporating legumes into Nutrient Film Technique (NFT) systems offers a sustainable cultivation model that supports both nutritional quality and ecological balance. This method also addresses food safety concerns, particularly the health risks associated with nitrate overaccumulation, as noted by Parks al. [62] and Wu et al. [63]. Combining moderate nitrogen fertilization with biological nitrogen fixation presents a viable approach to stabilizing crop yields while adhering to regulatory standards in controlled-environment agriculture. These findings emphasize the potential of legume-assisted hydroponics to promote reduced-input systems in line with the principles of circular agriculture.

## 4. Materials and Methods

**Figure 4:**
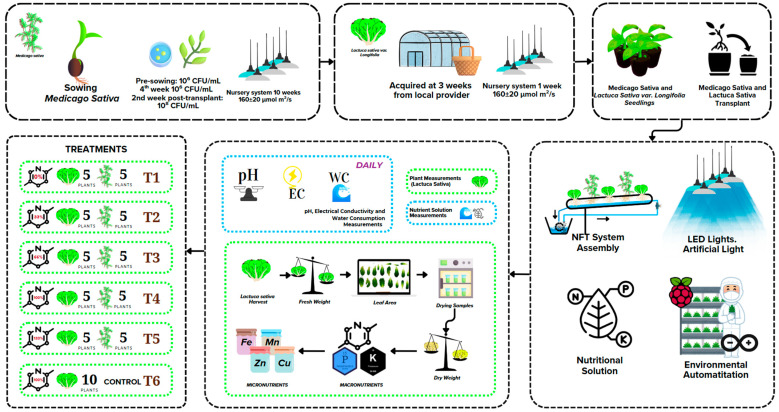
General scheme of the experiment for growing.

Figure 4 illustrates the experimental design used to assess the effects of varying nitrogen concentrations on the growth of romaine lettuce (*Lactuca sativa* var. *longifolia*) intercropped with alfalfa (*Medicago sativa*) in a vertical hydroponic Nutrient Film Technique (NFT) system. The experiment was conducted under artificial LED lighting (270 ± 40 μmol m^−2^ s^−1^) with fully automated environmental control. The study took place in the Artificial Lighting Laboratory at the Tecnológico Nacional de México—Campus Pabellón de Arteaga, located in Aguascalientes, Mexico (22.1464° N, 102.2591° W). Six treatments were established, representing nitrogen levels of 0%, 33%, 66%, 100%, and 133%. Treatments IC-N_0%_ to IC-N_133%_ consisted of co-cultures of lettuce and alfalfa, with five plants of each species per treatment. The control treatment, CK-N_100%_, was a lettuce monoculture containing 10 plants supplied with 100% of the standard nitrogen concentration. *Medicago sativa* was inoculated with nitrogen-fixing bacteria (*Rhizobium*) at three stages: pre-planting (10^6^ CFU/mL), two weeks after transplanting (10^8^ CFU/mL), and four weeks after transplanting (10^6^ CFU/mL). *Lactuca sativa* seedlings were purchased commercially at three weeks of age and acclimated for one week before transplantation into NFT channels. Throughout the experiment, daily monitoring was performed for pH, electrical conductivity (EC), and water consumption (WC) of nutrient solutions. At the end of the crop cycle, various metrics were measured for lettuce, including leaf number, leaf area (determined via digital scanning), fresh mass, dry mass (measured after oven-drying), root mass, and tissue nutrient content. During the harvest, three plants per treatment were selected for analysis. To eliminate positional bias, plant selection was randomized within each treatment group using a simple random sampling method. Fully expanded leaves were collected from these plants for nutrient and ion content analysis. Water use efficiency (WUE) was calculated as the ratio of dry mass (in grams) to the volume of nutrient solution consumed (in liters). This systematic approach ensured representative and unbiased data collection for evaluating the physiological and nutritional performance of lettuce under different nitrogen regimes in association with alfalfa. Further research could apply these findings to commercial multi-cropping systems with longer cycles.

### 4.1. Plant Material

Three-week-old seedlings of lettuce (*Lactuca sativa* L. *Longifolia*, LS) were sourced from a local provider in Asientos, Aguascalientes, Mexico. These plants were kept in a nursery system for one week under light conditions of 160 ± 20 µmol m^2^ s^−1^ before being transplanted into the hydroponic system. Certified seeds of *Medicago sativa* (MS) variety San Miguelito, which had germination and purity rates of 90% and 98%, respectively, were sown in a 60-cavity black plastic seedbed measuring 20 cm × 60 cm × 5 cm. The MS plants were transplanted into the same hydroponic system at the tenth week, achieving a height of 25 ± 5 cm after their first cut.

Before sowing, the MS seeds were immersed in a suspension of Rhizobium at a concentration of 10^6^ colony-forming units (CFUs)/mL^−1^. The MS seedlings were also exposed to Rhizobium again in the second and fourth weeks after germination at the same concentration. Finally, in the second week after transplanting, Rhizobium was supplied at a concentration of 10^8^ CFUs/mL^−1^ to ensure adequate colonization and symbiosis with the plants.

### 4.2. Biofertilizers Used in the Experiment

Five experimental conditions were established based on nutrient solution balances to modify nitrate levels (see Table 5). Municipal tap water from TecNM | Campus Pabellon de Arteaga was analyzed to determine its anionic and cationic composition. This analysis was conducted by Fertilab | Agricultural Laboratory, located in Cd. Industrial de Celaya, Celaya, Gto, Mx. Based on these results, a suitable nutrient solution balance for both plant species *Lettuca Sativa* (LS) and *Medicago sativa* (MS) was formulated. All solutions were balanced to maintain an electrical conductivity (EC) of 2.0 ± 0.5 mS cm^−1^ and a pH of 6.0 ± 0.5.

### 4.3. Plant Nutrition

Table 6 summarizes the nutrient solution formulations corresponding to each nitrate proportion used in the experiment. All solutions were prepared in 50 L containers, and the nutrient composition was carefully adjusted to maintain cationic balance [64], ensuring that variations in nitrate levels did not alter the electrical neutrality of the solution.

### 4.4. Plant Growing Conditions

An automatic vertical farming system utilizing Internet of Things technology was implemented in a controlled environment. The multilevel hydroponic structure utilizing the Nutrient Film Technique (NFT) was installed indoors on a rack measuring 1.955 m × 0.609 m × 1.981 m, within a temperature-controlled cold room. Each level of the system contains three troughs, each 1.8 m long, with fifteen holes spaced evenly for placing the plants. The experiment involved three replicates, with five lettuce and five alfalfa plants for each of the treatments (IC-N_0%_, IC-N_33%_, IC-N_66%_, IC-N_100%_, and IC-N_133%_), and ten lettuce plants for the control group (CK-N_100%_).

Nutrient solutions were stored independently in each gutter. A submersible pump facilitated the transfer of the nutrient solution through a half-inch CPVC pipe. The flow was regulated by a 5/8-inch PVC hydraulic valve, maintaining a 1% slope for the gutters and a water flow rate of 2.0 ± 0.2 L per minute. The temperature was set at 20 ± 2 °C, with a relative humidity of 60 ± 10%, and the carbon dioxide (CO2) concentration was established at 1100 ± 300 ppm.

#### Light System Characteristics

**Figure 5:**
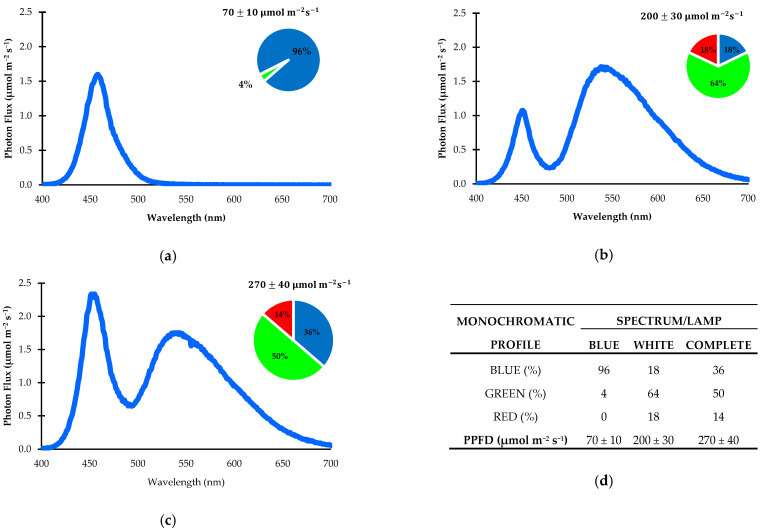
Distribution of photosynthetic photon flux density (PPFD) across different lighting treatments: (**a**) white LED lamps; (**b**) blue LED lamps; (**c**) both light sources combined; (**d**) summary of spectral composition and proportions of light recipes for each spectrum. All values are expressed in nanometers (nm) within the electromagnetic spectrum.

The artificial light environment was established using a combination of two LED light sources. One white LED emitted a photosynthetic photon flux density (PPFD) of 200 ± 30 µmol m^−2^ s^−1^, while a blue LED produced a PPFD of 70 ± 10 µmol m^−2^ s^−1^. Light intensity was measured with a LightScout Solar Electric Quantum Meter from Spectrum Technologies, Inc., Aurora, IL, USA. The emission spectra of the LEDs are shown in Figure 5, recorded using a RedTide spectrophotometer from Ocean Optics, Inc., Dunedin, FL, USA. The photoperiod was set to 16 h per day, resulting in a total PPFD of 270 ± 40 µmol m^−2^ s^−1^ and a daily light integral (DLI) of 15.55 ± 2.31 mol photons m^−2^ d^−1^ [52].

### 4.5. Measurements of Plant Growth Parameters

#### 4.5.1. Leaf Area (LA) and Number of Leaves (NLs)

Leaf area (LA) was determined through digital image analysis utilizing ImageJ software version 1.54g. On day 30 after transplanting, the leaf blades from selected plants were separated and photographed. Images were captured using an Apple iPhone 14 Pro equipped with a 48 MP wide-angle camera (f/1.78 aperture, Sony sensor; Apple Inc., Cupertino, CA, USA) at a distance of 1 m. Additionally, the leaves were categorized into three groups: small, medium, and large, based on the largest and smallest leaf areas observed across all treatments.

#### 4.5.2. Aerial Fresh and Dry Weight (AFW, ADW)

The aerial fresh weight (AFW) was measured 30 days after transplanting (DAT). At harvest, the shoot and root biomass were separated and weighed individually using an OHAUS Scout® balance (OHAUS Corporation, Parsippany, NJ, USA). Following fresh weight determination, the samples were placed in paper bags and dried in a forced convection oven (BINDER GmbH, Tuttlingen, Germany) at 70 °C for 72 h. After drying, the samples were weighed again to obtain dry weight.

#### 4.5.3. Elemental Nutrient Analysis

For the analysis of plant tissue nutrients, samples were dried, packaged in kraft paper bags, and sent to Fertilab Agricultural Laboratory located in Cd. Industrial de Celaya, Guanajuato, Mexico, 38010. The laboratory is accredited as “Laboratorio de Ensayo EMA (SA-1359-044/21)”. The analysis included the determination of total nitrogen using the Dumas method (accredited). Additionally, the elemental concentrations of boron (B), phosphorus (P), potassium (K), sodium (Na), calcium (Ca), magnesium (Mg), sulfur (S), iron (Fe), zinc (Zn), manganese (Mn), copper (Cu), molybdenum (Mo), and nickel (Ni) were measured through acid digestion followed by ICP-OES analysis.

#### 4.5.4. Specific Leaf Area (SLA)

Specific leaf area (SLA) was calculated using Equation (1), based on leaf area and aerial dry mass, following the method described by Pennisi et al. (2019) [65].

(1) SLA=LALMdr

where SLA represents the specific leaf area (cm^2^/g DW), LA is the leaf area (cm^2^), and M_dr_ is the aerial dry weight (g).

#### 4.5.5. Water Use Efficiency (WUE)

Equation (2) presents the calculation of water use efficiency (WUE), a key indicator of resource utilization in controlled environments. WUE was determined by dividing the total plant fresh weight (g DW) by the net volume of water consumed (L H_2_O) during the crop cycle. The net water volume was calculated by subtracting the volume of water collected through condensation by the mini-split unit (operating in “dry” mode) from the total water supplied. This collected water, assumed to originate from evapotranspiration and system humidity, was evenly distributed among the six treatments; hence, it was divided by 6 to estimate its contribution per treatment:

(2) $$WUE=\frac{Plant\;Dry\;Weight\;(g\;DW)}{Water\;Volume\;Used\left(L\;H_{2}O\right)-\frac{COLLECTED\;WATER\left(L\;H_{2}O\right)}{6}}$$

### 4.6. Measurements of Water Consumption (WC), pH, and Electrical Conductivity (EC)

#### 4.6.1. Water Consumption (WC)

Water consumption was monitored weekly throughout the experiment. Each treatment used 50 L-capacity basins, which were initially filled with 47.5 L of water. To track the remaining water volume over time, a simulation was designed in Python 3.10 using the geometric dimensions of the basin. This approach established a direct relationship between the liquid level (height) and the actual volume, allowing for accurate estimation of weekly water consumption based on changes in height.

#### 4.6.2. pH and EC

The pH and electrical conductivity (EC) were measured daily for all six nutrient solutions before the start of the daily light integral (DLI) using an AQUASEARCHER™ AB33M1-F benchtop meter (OHAUS Corporation, Parsippany, NJ, USA). The target pH value was set within the range of 6.0 ± 0.5. If necessary, pH adjustments were made by adding nitric acid or sulfuric acid, depending on the specific treatment, to avoid altering the nitrate (NO_3_^−^) concentration. Sodium hydroxide ACS (Fermont) was used when it was necessary to increase the pH. The target EC value was set between 1.8 and 2.5 mS cm^−2^, and no adjustments to the EC were required throughout the experiment.

### 4.7. Energy Consumption

For each treatment, energy consumption was measured using a STEREN electricity consumption meter (Wattmeter-W) HER-432 (Electrónica Steren S.A. de C.V., Mexico City, Mexico). This equipment operates at 120 V AC and a frequency of 60 Hz, with a minimum current measurement of 1 mA up to 15 A. Power consumption readings are independent of the measured values. Electricity consumption was divided into four main categories: lamps, pumps, CO_2_ systems, and a mini-split HVAC system. Additionally, consumption from the automated environment, which includes Raspberry Pi 4B, Arduino, ESP32CAM sensors and various actuators, was also measured using the same STEREN electricity consumption meter.

### 4.8. Statical Analysis

An analysis of variance (ANOVA) was conducted to identify significant differences among treatments regarding various physiological and growth-related variables. When significant differences were found (*p* < 0.05), Tukey’s HSD (honestly significant difference) test was used for mean comparisons between treatments, maintaining a significance level of 5% (95% confidence). All statistical analyses were performed using MINITAB 20 software, with a confidence level of 95% (α = 0.05).

## 5. Conclusions

This study evaluated the effects of different nitrate levels in a vertical hydroponic Nutrient Film Technique (NFT) system that intercropped lettuce (*Lactuca sativa*) with alfalfa (*Medicago sativa*). Among the intercropping treatments, the variant IC-N_66%_ demonstrated improved water use efficiency (WUE) and a slightly larger leaf area compared to the other treatments. However, the control treatment (CK-N_100%_), which consisted of a fully fertilized monoculture of lettuce, produced the highest overall fresh and dry biomass and nutrient uptake. Although IC-N_66%_ did not surpass the control in total yield, it showed more efficient resource use and moderate reductions in nitrate accumulation, suggesting potential benefits for sustainability in controlled environments. The findings suggest that intercropping nitrogen-fixing legumes with moderate nitrate supply can enhance water and nutrient efficiency without significantly sacrificing crop performance. To further this understanding, future research should explore longer cultivation periods, a variety of crop combinations, and indicators of postharvest quality. Overall, this study supports the creation of sustainable, low-input vertical farming models that align with global goals for food safety, resource efficiency, and environmental resilience.

## Figures and Tables

**Table 1:** Variation in nutrient solution pH across treatments over the crop cycle. Values are expressed as mean ± standard deviation. Different letters indicate significant differences (Tukey, *p* ≤ 0.05).

Treatments	DAT 0	DAT 07		DAT 14		DAT 21		DAT 30
		Initial	Final	Initial	Final	Initial	Final	
IC-N_0%_	6.02 ± 0.03 ^a^	6.87 ± 0.03 ^c^	5.99 ± 0.04 ^a^	6.88 ± 0.02 ^c^	6.02 ± 0.02 ^a^	6.21 ± 0.02 ^c^	6.16 ± 0.01 ^a^	6.06 ± 0.03 ^b^
IC-N_33%_	6.00 ± 0.02 ^a^	7.36 ± 0.04 ^a^	5.94 ± 0.01 ^a^	7.33 ± 0.04 ^a^	6.06 ± 0.04 ^a^	6.85 ± 0.03 ^a^	6.06 ± 0.02 ^b^	6.46 ± 0.03 ^a^
IC-N_66%_	6.01 ± 0.01 ^a^	6.92 ± 0.02 ^c^	5.98 ± 0.03 ^a^	6.99 ± 0.04 ^b^	6.03 ± 0.03 ^a^	6.85 ± 0.03 ^a^	6.01 ± 0.04 ^b^	6.13 ± 0.02 ^b^
IC-N_100%_	5.99 ± 0.04 ^a^	6.25 ± 0.02 ^d^	6.04 ± 0.02 ^a^	6.24 ± 0.01 ^d^	6.09 ± 0.03 ^a^	6.25 ± 0.03 ^c^	6.06 ± 0.03 ^b^	6.09 ± 0.02 ^b^
IC-N_133%_	6.01 ± 0.04 ^a^	7.15 ± 0.04 ^b^	6.00 ± 0.01 ^a^	6.90 ± 0.04 ^c^	6.04 ± 0.01 ^a^	6.59 ± 0.02 ^b^	6.03 ± 0.01 ^b^	6.13 ± 0.03 ^b^
CK-N_100%_	6.00 ± 0.01 ^a^	6.04 ± 0.03 ^e^	6.04 ± 0.02 ^a^	5.37 ± 0.01 ^e^	5.90 ± 0.03 ^a^	5.50 ± 0.02 ^d^	5.90 ± 0.02 ^b^	5.30 ± 0.01 ^c^

**Table 2:** Effect of treatments on leaf area, specific leaf area (SLA), leaf number, fresh mass, dry mass, root biomass, and water use efficiency (WUE) in lettuce plants at 30 DAT. Different letters indicate significant differences (Tukey, *p* ≤ 0.05). Bold letters indicate the most relevant values among treatments.

Treatments						
	IC-N_0%_	IC-N_33%_	IC-N_66%_	IC-N_100%_	IC-N_133%_	CK-N_100%_
Plant Parameters	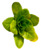	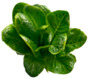	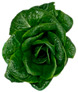	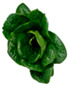	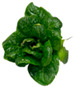	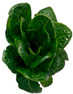
Leaf number	16.50 ± 2.66 ^b^	**29.00 ± 3.58 ^a^**	**30.33 ± 2.33 ^a^**	**29.00 ± 2.28 ^a^**	24.83 ± 3.19 ^b^	28.67 ± 5.24 ^ab^
Leaf area (cm^2^)	344.90 ± 86.50 ^c^	1297.14 ± 263.00 ^ab^	**1512.62 ± 179.40 ^a^**	1041.95 ± 165.30 ^b^	1173.23 ± 170.40 ^b^	1357.75 ± 233.90 ^ab^
Fresh mass (g)	17.93 ± 3.98 ^c^	88.30 ± 26.80 ^ab^	**104.57 ± 12.04 ^ab^**	83.55 ± 14.48 ^b^	98.79 ± 17.52 ^ab^	**114.60 ± 13.27 ^a^**
Dry mass (g)	5.09 ± 1.32 ^b^	7.63 ± 2.10 ^ab^	**8.95 ± 1.53 ^a^**	7.63 ± 1.36 ^ab^	7.55 ± 2.93 ^ab^	**9.90 ± 1.86 ^a^**
Root mass (g)	19.27 ± 2.11 ^c^	28.58 ± 6.15 ^b^	33.93 ± 3.08 ^ab^	34.90 ± 5.20 ^ab^	**37.97 ± 4.09 ^a^**	33.23 ±6.36 ^ab^
SLA (cm^2^ g^−1^)	70.49 ± 21.39 ^b^	178.20 ± 46.80 ^ab^	170.36 ± 15.82 ^ab^	138.17 ± 19.25 ^ab^	**202.20 ± 15.41 ^a^**	138.62 ± 19.63 ^ab^
WUE (gDW L^−1^)	1.57 ± 0.43 ^b^	2.24 ± 0.62 ^ab^	**2.81 ± 0.48 ^a^**	2.39 ± 0.43 ^ab^	2.52 ± 0.99 ^ab^	**3.26 ± 0.62 ^a^**

**Table 3:** Concentrations of macronutrients and micronutrients in lettuce leaves at 30 DAT under different nitrate treatments. The values are expressed as means ± standard deviations (*n* = 3). Different letters indicate statistically significant differences (*p* ≤ 0.05) based on Tukey’s test. Bolded values highlight nutrient levels that fall significantly outside the standard literature range.

Nutrients (mg g^−1^ DW)		Treatments						Literature Ranges		
		IC-N_0%_	IC-N_33%_	IC-N_66%_	IC-N_100%_	IC-N_133%_	CK-N_100%_	Min	Max	References
Macronutrients	Nitrogen (N)	12.80 ± 0.77 ^c^	**40.40 ± 2.44 ^ab^**	38.60 ± 2.33 ^b^	**43.80 ± 2.65 ^a^**	**41.30 ± 2.50 ^ab^**	**40.40 ± 2.44 ^ab^**	24	40	[12,30,31]
	Phosphorus (P)	3.80 ± 0.23 ^d^	**10.10 ± 0.61 ^b^**	8.60 ± 0.52 ^c^	**10.90 ± 0.66 ^ab^**	**11.30 ± 0.67 ^a^**	**11.20 ± 0.68 ^a^**	6	9.5	[32,33,34]
	Potassium (K)	37.30 ± 2.20 ^c^	**85.20 ± 5.16 ^a^**	77.20 ± 4.67 ^b^	79.00 ± 4.78 ^ab^	82.60 ± 5.00 ^ab^	77.60 ± 4.70 ^b^	55	85	[32,35]
	Calcium (Ca)	6.30 ± 0.38 ^c^	13.20 ± 0.80 ^a^	11.70 ± 0.71 ^b^	11.10 ± 0.67 ^b^	11.30 ± 0.68 ^b^	11.80 ± 0.71 ^b^	9	14	[36,37]
	Magnesium (Mg)	2.00 ± 0.12 ^c^	3.80 ± 0.23 ^a^	3.30 ± 0.19 ^b^	3.70 ± 0.22 ^a^	3.70 ± 0.23 ^a^	3.10 ± 0.18 ^b^	2.5	4	[38,39]
	Sulfur (S)	1.50 ± 0.10 ^d^	**2.60 ± 0.16 ^bc^**	2.40 ± 0.14 ^c^	**3.00 ± 0.18 ^a^**	**2.90 ± 0.17 ^a^**	**2.80 ± 0.18 ^ab^**	1.8	2.6	[30,40]
Micronutrients	Iron (Fe)	19.05 ± 1.18 ^d^	**39.10 ± 2.36 ^b^**	34.80 ± 2.10 ^c^	**39.20 ± 2.37 ^b^**	**42.00 ± 2.54 ^b^**	**48.30 ± 2.92 ^a^**	24	35	[38,41,42]
	Zinc (Zn)	65.30 ± 3.95 ^c^	**241.00 ± 14.59 ^ab^**	**262.00 ± 15.86 ^a^**	**244.00 ± 14.77 ^ab^**	**223.00 ± 13.50 ^b^**	**234.00 ± 14.17 ^b^**	120	190	[43,44]
	Manganese (Mn)	239.00 ± 14.47 ^d^	374.00 ± 22.65 ^c^	**477.00** ± 28.9 **^a^**	419.00 ± 25.37 ^b^	373.00 ± 22.59 ^c^	367.00 ± 22.22 ^c^	310	450	[30,42]
	Copper (Cu)	3.16 ± 0.19 ^d^	9.97 ± 0.60 ^c^	9.63 ± 0.58 ^c^	**11.50 ± 0.69 ^b^**	**11.20 ± 0.67 ^b^**	**12.90 ± 0.78 ^a^**	6	10	[30,42]
	Boron (B)	30.60 ± 1.85 ^d^	**42.50 ± 2.57 ^a^**	36.40 ± 2.20 ^b^	**42.30 ± 2.56 ^a^**	34.80 ± 2.10 ^bc^	31.50 ± 1.90 ^cd^	28	40	[42,45]
	Nickel (Ni)	**5.52 ± 0.33 ^a^**	0.37 ± 0.02 ^c^	0.16 ± 0.01 ^c^	0.22 ± 0.01 ^c^	0.20 ± 0.01 ^c^	**0.64 ± 0.04 ^b^**	0.15	0.35	[43,46,47]
	Molybdenum (Mo)	**0.10 ± 0.006 ^b^**	**0.12 ± 0.007 ^a^**	0.06 ± 0.003 ^d^	0.05 ± 0.003 ^e^	0.09 ± 0.005 ^c^	**0.10 ± 0.005 ^b^**	0.04	0.09	[42,48]

**Table 4:** Monthly energy consumption by subsystems in the vertical farming system.

Subsystem	Energy Consumption (kWh month^−1^)	Percentage of Total (%)
Temperature	206.1	33.58
CO_2_	2.7	0.45
Lights	293.8	47.88
Irrigation	99.7	16.25
System	11.3	1.84

**Table 5:** Distribution of treatments and relative percentage of NO_3_^−^-used.

Treatment Code	NO_3_^−^ (% of Control)	Intercropping	Plants (% Individuals)
IC-N_0%_	0	Yes	Lettuce (50%) and alfalfa (50%)
IC-N_33%_	33	Yes	Lettuce (50%) and alfalfa (50%)
IC-N_66%_	66	Yes	Lettuce (50%) and alfalfa (50%)
IC-N_100%_	100	Yes	Lettuce (50%) and alfalfa (50%)
IC-N_133%_	133	Yes	Lettuce (50%) and alfalfa (50%)
CK-N_100%_	100	No	Lettuce (100%)

**Table 6:** Distribution of treatments and relative nitrate (NO_3_^−^) supply used in each experimental group.

Ions (mEq L^−1^)		Treatments				
		IC-N_0%_	IC-N_33%_	IC-N_66%_	IC-N_100%_	CK-N_100%_
Macronutrients	KNO_3_	0.00	0.00	0.00	3.00	6.50
	Ca(NO_3_)_2_	0.00	4.00	8.00	9.00	9.00
	HNO_3_	0.00	0.90	0.90	0.90	0.90
	KH_2_PO_4_	7.00	6.50	4.50	1.00	0.50
	K_2_SO_4_	0.00	0.50	2.50	3.00	0.00
	CaSO_4_	9.00	5.00	1.00	0.00	0.00
	MgSO_4_·7H_2_O	4.00	4.00	4.00	4.00	3.50
	H_2_SO_4_	0.90	0.00	0.00	0.00	0.00
Micronutrients	FeSO_4_	0.1430	0.1430	0.1430	0.1430	0.1430
	CuSO_4_	0.0194	0.0194	0.0194	0.0194	0.0194
	MnSO_4_	0.0031	0.0031	0.0031	0.0031	0.0031
	ZnSO_4_	0.0100	0.0100	0.0100	0.0100	0.0100
	Na_2_ [B_4_O_5_ (OH)_4_]	0.0065	0.0065	0.0065	0.0065	0.0065
	Na_2_MoO_4_	0.0030	0.0030	0.0030	0.0030	0.0030

## Data Availability

Data are contained within the article.
